# Do corticosteroids reduce the mortality of influenza A (H1N1) infection? A meta-analysis

**DOI:** 10.1186/s13054-015-0764-5

**Published:** 2015-12-01

**Authors:** Yi Zhang, Wenjie Sun, Erik R Svendsen, Song Tang, Raina C MacIntyre, Peng Yang, Daitao Zhang, Quanyi Wang

**Affiliations:** 1 Beijing Center for Disease Prevention and Control (CDC), No. 16 He Pingli Middle Street, Dongcheng District, Beijing, 100013 China; 2 School of Public Health and Tropical Medicine, Tulane University, 1440 Canal Street, Suite 2100, New Orleans, LA 70112 USA; 3 School of Food Science, Guangdong Pharmaceutical University, Zhongshan, 528458 China; 4 School of Environment and Sustainability, University of Saskatchewan, 44 Campus Drive, Saskatoon, SK S7N 5B3 Canada; 5 School of Public Health and Community Medicine, The University of New South Wales, Sydney, NSW 2052 Australia

**Keywords:** Corticosteroid, Influenza, Oseltamivir, Corticosteroid Treatment, Antiviral Treatment

## Abstract

**Introduction:**

Corticosteroids are used empirically in influenza A (H1N1) treatment despite lack of clear evidence for effective treatment. This study aims to assess the efficacy of corticosteroids treatment for H1N1 infection.

**Methods:**

Systematic review and meta-analysis were used to estimate the efficacy of corticosteroids for the prevention of mortality in H1N1 infection. Databases searched included MEDLINE, EMBASE, PubMed, Cochrane Central Register of Controlled Clinical Trials and so on, and bibliographies of retrieved articles, from April 2009 to October 2014. We included both cohort studies and case-control studies reported in English or Chinese that compared treatment effects between corticosteroids and non-corticosteroids therapy in inpatients with H1N1 virus infection. Cohort studies employed mortality as outcome, and case-control studies employed deaths as cases and survivors as controls; both were assessed in this meta-analysis.

**Results:**

In total twenty-three eligible studies were included. Both cohort studies (nine studies, n = 1,405) and case-control studies (14 studies, n = 4,700) showed a similar trend toward increased mortality (cohort studies relative risk was 1.85 with 95% confidence interval (CI) 1.46 to 2.33; case-control studies odds ratio was 4.22 with 95% CI 3.10 to 5.76). The results from both subgroup analyses and sensitive analyses were consistent with each other, showing that steroid treatment is associated with mortality. However, considering the fact that corticosteroids were tend to be used in sickest case-patients and heterogeneity was observed between studies, we cannot make a solid conclusion.

**Conclusions:**

Available evidence did not support the use of corticosteroids as standard care for patients with severe influenza. We conclude that further research is required.

**Electronic supplementary material:**

The online version of this article (doi:10.1186/s13054-015-0764-5) contains supplementary material, which is available to authorized users.

## Introduction

Novel influenza A (H1N1) spread around the world in spring 2009. Although influenza A (H1N1) infection has a mild clinical course, the pandemic virus is capable of leading to severe illness, requiring hospitalization. As an example, the hospital admission rate for children with 2009 H1N1 influenza was twofold the rate for seasonal influenza [1]. Additionally, approximately 9 to 31% of hospitalized patients were admitted to an ICU, where 14 to 46% of patients died [2-5]. The disease caused 284,500 deaths globally [6,7]. Accordingly, there is an increasing need for the development of an effective therapy and treatment to improve upon the prognosis of severe cases.

In severe influenza infectious cases, cytokine dysregulation was observed in patients [8] and corticosteroids had been proven to be able to reduce systemic inflammation by inhibition of proliferation and cytokine production [8-11]. Previous meta-analyses of patients with acute long injury and acute respiratory distress syndrome indicated that prolonged glucocorticoid treatment is safe and is associated with significant reductions in markers of systemic inflammation, multiple organ dysfunction score, duration of mechanical ventilation, and ICU length of stay [11,12]. Moreover, Nie and colleagues’ study showed that the use of corticosteroids was associated with improved mortality in severe community-acquired pneumonia (CAP) [13]. According to the above accounts, corticosteroids were used in 40 to 53% of patients with confirmed or probable H1N1 virus infection with various dose regimens [14-16], and about 22% of inpatient children with H1N1 were treated with corticosteroids [17]. Corticosteroids were empirically used as a preferred or lifesaving treatment and were observed in more than 50% of the severe patients, including acute respiratory distress syndrome, during the pandemic influenza in 2009 [4,18].

Although corticosteroids are widely used, the effect of corticosteroids on pandemic A (H1N1) influenza patients has not been studied adequately and, thus, is still controversial. For example, in several studies a remarkable effect was observed of early treatment with oseltamivir and steroids for patients with severe pneumonia in preventing disease progression [19-21]. Additionally, a number of clinical case series and case reports have shown that patients with severe respiratory complications, pneumonia, improved after the use of corticosteroids [22-24]. However, the USA Centers for Disease Control and Prevention does not recommend the use of corticosteroids as a primary medicine for H1N1 infection, with the exception that a reasonable dose is indicated for a specific reason; for example, pulmonary obstruction or septic shock [25]. Moreover, World Health Organization guidelines for Pharmacological Management of Pandemic Influenza A (H1N1) 2009 and other Influenza Viruses recommend that systemic corticosteroids should not be administrated to patients who have severe or progressive clinical illness unless in some exceptional circumstances [26,27]. Severe influenza treatment guidelines for Korea also indicate that systemic corticosteroid administration should not be performed for the treatment of severe influenza patients unless the therapeutic effect has already been proven [28]. None of the guidelines above recommend systematic corticosteroid use regularly with H1N1 infection. However, lack of clinical evidence makes these recommendations or guidelines unconvincing.

Until now, many studies involving the treatment of severe H1N1 cases have been published, but the results are inconsistent, which could be due to insufficient sample sizes, complicated clinical status, or study design. To our knowledge, there has been no systematic literature review evaluating the benefit of corticosteroids to severe H1N1 infection. A principal resource for the optimal clinical therapy of influenza A (H1N1) patients and directions for future research are warranted.

We therefore conducted the present study to determine whether corticosteroids can treat severe H1N1 infection. To clarify the association of corticosteroids with H1N1 mortality taking into account clinical status and study design, we examined the associations in larger, prospective cohort studies in global settings, using existing literature, and assessed the effect of corticosteroids treatment on mortality through meta-analysis.

## Methods

### Search strategy and selection criteria

We conducted a comprehensive literature search both for English-language and Chinese-language articles examining the effect of corticosteroid treatment in influenza A (H1N1) published up until October 2014. Electronic databases searched included: MEDLINE, EMBASE, PubMed, Cochrane Central Register of Controlled Clinical Trials, University of Saskatchewan Library System, China National Knowledge infrastructure, Wan fang Data, and CBM disc. We contacted article authors for further information or clarification when necessary. No attempt was made to include unpublished data. All searches were executed independently by two skilled researchers. The search strategy consisted of the terms (‘A (H1N1)’ or ‘A/H1N1’ or ‘influenza’ or ‘viral pneumonitis’) and (‘corticosteroids’ or ‘steroids’) as medical subject-heading key words. In addition, the reference lists of retrieved original articles and of relevant systematic reviews were manually checked. No ethics board approval was deemed necessary for a meta-analysis of previously published studies.

### Eligibility criteria

Because there was no randomized trial available, we included both cohort studies and case–control studies. We included cohort studies fulfilling the following selection criteria: enrolled patients had confirmed, probable, or suspected influenza A (H1N1); all of the subjects were inpatient, or admitted to the ICU, or critically ill; corticosteroid treatment was compared with noncorticosteroid treatment within the cases; and data about hospital mortality were accessible. For case–control studies, the inclusion criteria were that: enrolled patients had confirmed, or had probable or were suspected of having influenza A (H1N1); all of the subjects were inpatient, or admitted to the ICU, or critically ill; deaths were cases and survivors were controls; and the numbers of patients who received or did not receive steroid treatment were presented in two groups. There were no restrictions on studies with respect to age groups.

Studies were excluded if they: included seasonal influenza infection cases; were *in vitro* tests, animal experiments, case studies, case series, and review or letter articles; and targeted special crowds, such as pregnant women and patients with HIV infections.

Additionally, confirmed influenza A (H1N1) cases were defined as an acute respiratory illness with laboratory confirmation by real-time PCR or viral culture. Corticosteroid treatment was defined as: systemic corticosteroid use; and nonstandardized corticosteroid use, which was decided by the attending physician and was regardless of type, dosage, and frequency of administration.

### Data extraction

All full articles were reviewed for the selection and exclusion of publications with predefined inclusion criteria by two researchers independently. We also contacted the corresponding author of 12 studies by email to ask for additional details. However, only three authors responded. For both case–control and cohort studies, the following information was collected for each study: first author, year of publication, country or origin, study design, inclusion/exclusion criteria, participant demographics, sample size, antiviral treatment, and corticosteroid dose, formulation, and duration. For case–control studies, we collected information about the numbers of patients treated with steroids in each group. With regard to cohort studies, information about the number of patients who died in each group and other clinical outcomes were collected. Disagreements were resolved by consensus.

### Qualitative assessment

The Newcastle-Ottawa Scale scoring system was used to assess the methodology and quality of both cohort studies and case–control studies [29]. The Newcastle-Ottawa Scale assigns a maximum score of 4 for selection, 2 for comparability, and 3 for exposure (case–control studies) or outcome (cohort studies). Hence, a score of 9 is the highest possible and reflects the best quality. Two investigators independently assessed the risk of bias of each study. The detailed evaluation criteria are shown in Additional file 1. Inter-rater agreement was assessed using Cohen's kappa statistics and disagreements were resolved by consensus.

### Statistical analysis

We calculated the relative risk for death within cohort studies, while the odds ratio (OR) was used for case–control studies. Heterogeneity of treatment effects among studies was assessed by examining forest plots, and statistically using Cochran *Q* and *I* ^2^ statistics. If significant heterogeneity was seen (*P* <0.1 and *I* ^2^ > 30), a random-effects model was selected; otherwise, a fixed-effects model was used. Two-sided *P* <0.05 was considered to be statistically significant. If a significant heterogeneity was identified, subgroup analyses were carried out. Studies were categorized by sample size, by whether the subjects from two groups are comparable in terms of age and antiviral treatment, and by whether the studies included probable and suspected A (H1N1) cases. Sensitivity analysis excluded studies one by one to investigate the heterogeneity. Potential publication bias was assessed by Begg and Mazumdar’s rank correlation test [30] and by observing funnel plots. We attempted to summarize the corticosteroids’ effect on other clinical outcomes by describing the results from studies. All statistical analysis was performed using the Comprehensive Meta-Analysis V2 software (Biostat Inc., Englewood, NJ, USA) and Review Manager V5 software (Nordic Cochrane Center, Copenhagen, Denmark).

### Role of the funding source

The sponsor of this study had no role in the study design, data collection, data analysis, data interpretation, writing of the report, or decision to submit the paper for publication. The corresponding authors had full access to all data in the study and had final responsibility for the decision to submit the paper for publication.

## Results

### Study characteristics

**Figure 1:**
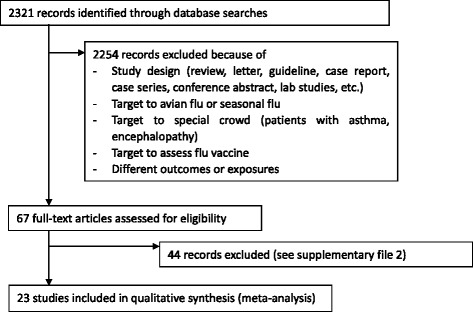
**Study identification, inclusion and exclusion.**

Of the 2,321 references screened, 23 studies were included in the final analysis (Figure 1). Fourteen studies were case–control studies [15,31-43], and nine studies were cohort studies [44-52]. In total, 6,105 subjects were analyzed, with 4,700 subjects in case–control studies and 1,405 subjects in cohort studies. Among these studies, seven studies were conducted in China [36,39,41-44,50], three in Spain [33,45,49], three in India [31,35,46], two in Korea [18,28], two in Argentina [15,40], and one study each was conducted in Mexico [37], Turkey [38], Saudi Arabia [48], France [51], and Finland [52], while the remaining study was multicenter and conducted in several countries (European Society of Intensive Care Medicine) [47]. The characteristics of the included studies are summarized in Table 1. Participants in all studies were inpatients. Eighteen studies only included patients in the ICU or critically ill cases [15,32-36,38-40,42,44,46-52], and three studies included children [35,36,40]. There was only one study that did not mention antiviral treatment use in patients [44]. Corticosteroid treatment varied among these studies, with most using methylprednisolone or hydrocortisone; doses varied from 80 to 320 mg daily. However, there were still several studies which did not describe the tapering doses in detail, or the precise duration of treatment. Two Spanish studies showed that corticosteroid administrations were not standardized and were decided by the attending physician without detailed data [33,49]. Another six studies did not provide any detailed information about dose, duration, and treatment mode [34,40,42-44,52].

**Table 1:** **Baseline characteristics of included studies**

**First author, year**	**Country**	**Study design**	**Population**	**Sample size**	**Mean/median age (years)**	**Female (** ***n*** **/%)**	**Antiviral**	**Corticosteroid doses and duration**
Rios, 2011 [15]	Argentina	Case–control	Confirmed influenza A (H1N1) and ARDS and mechanical ventilation and ICU	178	44	98/55.0	Treatment with oseltamivir was given to 98% of patients, with 60% receiving 300 mg/day. The frequency of use and doses were similar in both survivors and nonsurvivors	Corticosteroids were prescribed in 36% of patients for septic shock as 300 mg/day hydrocortisone
Chawla, 2013 [31]	India	Case–control	Confirmed influenza A (H1N1) cases and inpatient	77	40.88	33/42.9	No statistical difference between two groups	Steroids were administered for an average duration of 10.61 days
Hong, 2013 [32]	South Korea	Case–control	Confirmed influenza A (H1N1) cases and critical illnesses and adult	245	55.3	111/45.3	All patients received antiviral therapy	Dose equivalent (prednisolone) 75 mg/day
Jose, 2013 [33]	Spain	Case–control	Confirmed influenza A (H1N1) cases and requiring ICU admission and age ≥15 years	1,120	72	365/32.6	Not comparable between two groups (more dead patients use antiviral after 48 hours after hospital admission)	Corticosteroid use was not standardized and was decided by the attending physician
Jung, 2011 [34]	South Korea	Case–control	Confirmed influenza A (H1N1) cases and critical illnesses and requiring ICU admission and age ≥15 years	221	57	103/46.6	All patients received antiviral treatment, and the duration from symptom onset to initial antiviral treatment did not differ	No mentioned
Kinikar, 2012 [35]	India	Case–control	Confirmed influenza A (H1N1) cases and inpatient or admitted to the ICU and children	92	2.5	49/53.0	All patients received antiviral treatment	Short course of corticosteroids was administered to 21 children
Li, 2012 [36]	China	Case–control	Confirmed influenza A (H1N1) case sand critical illnesses and children and inpatient	1,137	4	390/34.3	Not comparable between two groups (more survival patients use antiviral within 48 hours of onset of illness)	Median duration of corticosteroids treatment was 6 days
Perez-Padilla, 2009 [37]	Mexico	Case–control	Confirmed influenza A (H1N1) cases and inpatient and pneumonia	18	38	9/50.0	None of the patients were given oseltamivir during the first 48 hours after the onset of symptoms	Corticosteroids were administered at the discretion of the attending physicians. Hydrocortisone at a dose of 300 mg/day or methylprednisolone at a dose of 60 mg/day
Sertogullarindan, 2011 [38]	Turkey	Case–control	Confirmed influenza A (H1N1) cases and requiring ICU admission and pneumonia	20	36	10/50.0	None of them had taken oseltamivir within 48 hours. Overall, patients received oseltamivir therapy at a dosage of 75 mg twice a day for 5 days	Not mentioned
Sun, 2010 [39]	China	Case–control	Confirmed influenza A (H1N1) cases and ICU	18	37	8/44.4	Oseltamivir 150 mg, twice daily	Methylprednisolone: 3 to 5 days, 1 to 2 mg/kg; or hydrocortisone 300 mg/day
Torres, 2012 [40]	Argentina	Case–control	Confirmed influenza A (H1N1) and pediatric ICU	142	19 months	86/60.0	All patients were treated with oseltamivir	No mentioned
Xi, 2010 [41]	China	Case–control	Confirmed influenza A (H1N1) cases and adult and inpatient	155	43	65/41.9	No statistical difference between two groups	There were 33.5% patients treated with systemic corticosteroids, daily dose of corticosteroids ranged from methylprednisolone 12 to 320 mg (or equivalent dose), with a median dose of 80 mg
Yu, 2011 [42]	China	Case–control	Confirmed influenza A (H1N1) cases and critical illnesses and inpatient	128	28.5	51/39.8	Not comparable between two groups (more survival patients used oseltamivir)	Not mentioned
Zhang, 2013 [43]	China	Case–control	Confirmed influenza A (H1N1) cases and severe or critical ill and ≥14 years old	2,151	34.0	1069/49.7	95.3% of patients received oseltamivir treatment	No mentioned
Zhang, 2011 [44]	China	Cohort	Confirmed influenza A (H1N1) cases and critical illnesses and inpatient	146	44.21	57/39.0	Not mentioned	High dose, high dose plus low dose, and low lose
Viasus, 2011 [45]	Spain	Cohort	Confirmed influenza A (H1N1) cases and pneumonia and inpatient	197	N/A	106/53.8	No statistical difference exists between steroid group and nonsteroid group	Seventeen (48%) patients received Corticosteroids at a daily dose above 300 mg hydrocortisone or its equivalent
Patel, 2013 [46]	India	Cohort	Confirmed influenza A (H1N1) cases and ICU	63	34	22/35.0	Patients without pneumonia were treated with oseltamivir, 75 mg p.o. twice daily, and those with pneumonia were treated with 150 mg p.o. twice daily. In pediatric patients, an appropriate weight-based dose of oseltamivir was used	Methylprednisolone 40 mg i.v. every 8 hours for first week followed by every 12 hours for second week and every 24 hours for third week were used for hypoxic patients with pulmonary opacities
Martin-Loeches, 2011 [47]	Europe	Cohort	Confirmed influenza A (H1N1) and ICU	220	43	113/51.4	All patients received antiviral therapy	Systemic corticosteroid use was considered when dosages equivalent to >24 mg/day methylprednisone or > 30 mg/day prednisone were given at ICU admission
Mady, 2012 [48]	Saudi Arabia	Cohort	Confirmed influenza A (H1N1) cases and admitted to the ICU and respiratory failure	86	40.8	22/28.0	Not comparable between two groups (more dead patients use antiviral after 48 hours after hospital admission)	Methylprednisolone 1 mg/kg per day for early phase ARDS, continued for 7 days
Diaz, 2012 [49]	Spain	Cohort	Confirmed influenza A (H1N1) cases and acute respiratory failure requiring ICU admission and pneumonia	372	43.4	167/44.9	All patients received antiviral therapy	Corticosteroids administered were not standardized and were decided by the attending physician
Chen, 2010 [50]	China	Cohort	Confirmed influenza A (H1N1) cases and critical illnesses	12	33.5	6/50.0	All patients received oseltamivir	Methylprednisolone 80 mg/day (five cases) or 320 mg/day (one case), median duration of corticosteroid treatment was 4.1 ± 1.5 days
Brun-Buisson, 2011 [51]	France	Cohort	Confirmed influenza A (H1N1) cases and requiring ICU admission and ARDS	208	45.5	105/50.5	Four patients did not receive antiviral therapy. Time from ARI to antiviral therapy use has no significant difference between two groups	Steroid therapy was initiated at a median daily dose equivalent to 270 (IQR, 200 to 400) mg hydrocortisone, and patients were treated for a median duration of 11 (IQR, 6 to 20) days
Linko, 2011 [52]	Finland	Cohort	Confirmed influenza A (H1N1) cases and admitted to the ICU	132	47 · 8	47/35.6	Oseltamivir was given to 96% patients. No statistical difference between two groups	Not mentioned
Kudo, 2012 [53]	Japan	Cohort	Confirmed influenza A (H1N1) cases and respiratory disorders and inpatient	89	80 cases < 15 years	44/49.4	All subjects were treated with antiviral agents, either oseltamivir or zanamivir	The dosage of corticosteroids was equivalent to methylprednisolone 1.0 to 1.5 mg/body weight (kg)/time, two to four times/day, in subjects under 15 years of age, and 40 to 80 mg/time, two to four times/day in those over 15 years of age. The median number of days from symptom onset to initiation of administration of systemic corticosteroids was 2.1 (range, 1 to 6). The median duration of systemic corticosteroid treatment was 5.2 days (range, 2 to 9)

ARDS, acute respiratory distress syndrome; ARI, acute respiratory infection; IQR, interquartile range; i.v., intravenously; p.o., *per os*.

### Qualitative assessment

**Figure 2:**
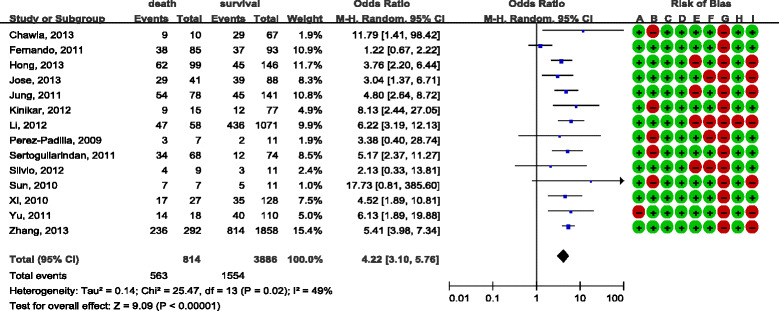
**Effect of corticosteroids on influenza A (H1N1) cases from case–control studies.** Diamond, overall estimate from the meta-analysis; square, point estimate of the result of each study; horizontal line that runs through the square and the width of the diamond represents the CI. Red dot, high risk of bias; green dot, low risk of bias; A to I, see Additional file 1. CI, confidence interval; M-H, Mantel-Haenszel.

**Figure 3:**
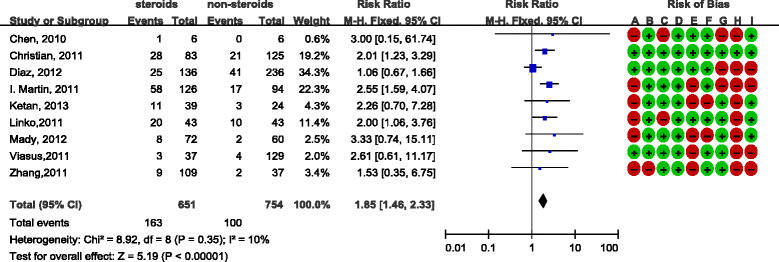
**Effect of corticosteroids on influenza A (H1N1) cases from cohort studies.** Diamond, overall estimate from the meta-analysis; square, point estimate of the result of each study; horizontal line that runs through the square and the width of the diamond represents the CI. Red dot, high risk of bias; green dot, low risk of bias; A to I, see Additional file 1. CI, confidence interval; M-H, Mantel-Haenszel.

There was consensus between reviewers with regard to the validity assessments (Cohen’s kappa was 58%). The overall quality of the included studies was moderate and the analyses are presented in Figure 2 and Figure 3. Most studies were retrospective and observational studies, and the most common bias was lack of comparability in terms of age or antiviral therapy between study groups. In addition, some studies did not provide detailed data with regard to corticosteroid use. Most of the study data were obtained either from the registration systems or by reviewing hospital records, and the response rate was not reported. They were therefore judged as high risk in this respect. Only a few studies demonstrated that they excluded steroid-use cases for underlining diseases, while other studies did not mention this at all.

### Primary outcome – mortality

#### Case–control studies

Significant heterogeneity was found for case–control studies (*I* ^2^ = 49%). The random-effects model was therefore used and the combined OR was 4.22 (95% CI = 3.10 to 5.76) (Figure 2).

Subgroup analysis was conducted to investigate the heterogeneity, and the results are shown in Additional file 2. The plots show that the sample size of studies, as well as comparability in terms of age and antiviral treatment, did not significantly influence the final mortality outcome. The heterogeneity was not statistically significant, after studies were categorized according to whether they enrolled suspected or probable cases, and the pooled OR was 5.05 (95% CI = 4.14 to 6.15) in studies that only enrolled confirmed cases, while the pooled OR was not statistical significant in the other group [15,39,40] (OR = 1.50, 95% CI = 0.87 to 2.58).

In sensitivity analysis, we found that heterogeneity was not examined after excluding Rios and colleagues’ study [15], and the result suggested that high mortality was associated with steroid treatment (OR = 4.97, 95% CI = 4.08 to 6.04).

#### Cohort studies

With regard to the nine cohort studies, we noted that there was no significant heterogeneity between studies (*I* ^2^ = 10%), so a fixed-effects model was used and the nine cohort studies had a relative risk of 1.85 (95% CI = 1.46 to 2.33), which suggested mortality was higher in patients who were given steroids (Figure 3). Subgroup analyses, as shown in Additional file 3, revealed that the relative risk was higher in studies of poor quality than that of the good quality studies. However, the difference was not statistically significant. Besides, the inclusion of probable and suspected cases did not significantly change the result, consistently showing steroid treatment was a risk factor of mortality.

### Other clinical outcomes

Zhang and colleagues’ study showed that corticosteroid treatment has a better therapeutic effect when compared with nonsteroid treatment [44]. Similarly, another Chinese study indicated that patients in steroid treatment groups have a shorter duration of fever and a shorter duration of inflammation [50]. In addition, Martin-Loeches and colleagues’ study showed that patients who received early corticosteroid therapy had hospital-acquired pneumonia more frequently than patients who did not [47].

Regarding length of hospitalization, Kudo and colleagues’ study demonstrated an increased length of hospital stay in patients with corticosteroid treatment when compared with the controls, despite no significant difference being found [53]. Also, Linko and colleagues study showed that the length of ICU and hospital stay was significantly longer in the patients treated with corticosteroids [52].

Regarding the duration of mechanical ventilation, Diaz and colleagues displayed that there was no difference between the corticosteroid and noncorticosteroid groups [49]. By contrast, Linko and colleagues’ study showed that patients treated with corticosteroids have significantly longer durations of mechanical ventilation [52]. Since the mean difference was not provided in Linko and colleagues’ study, we were not able to calculate the pooled effect. Viasus and colleagues’ study found that patients who received corticosteroid treatment needed significantly more time to reach clinical stability [45].

### Publication bias

In the present meta-analysis, no publication bias was observed between case–control studies and cohort studies using the Begg and Mazumdar rank correction test (*P* = 0.8 and *P* = 0.91). However, the funnel plots provided evidence of publication bias for both types of studies (Additional file 4).

## Discussion

During the 2009 influenza pandemic, the debate over whether to use corticosteroid treatment in severe influenza H1N1-infected patients resurfaced and was disputed by clinicians [26]. According to our review, corticosteroid administration is likely to increase mortality in patients with influenza A (H1N1), and the trend is consistent regardless of the quality as well as the sample size of studies. Apart from the studies included in this meta-analysis, there are many studies that refer to the steroids used and the outcomes, and most of them reported that corticosteroids have negative effects or no effect on H1N1 treatment. For example, Balaganesakumar and colleagues found that corticosteroid treatment would cause a higher risk of poor patient outcomes [54]. Other reports showed that patients who received corticosteroids were more likely to develop secondary bacterial pneumonia [47,55] or were associated with an increased risk of developing critical illness, with ICU admission, or had more prolonged ICU stays [17,55-57]. The possible explanation for the negative effectiveness of corticosteroids might be that corticosteroids could inhibit immune reactions. However, immune systems should be activated in order to eliminate the virus [58]. Altered immune reactions thus might lead to prolonged virus viremia [59] and delay viral clearance [60], and ultimately increase the risk of mortality.

Indeed, there are several studies that reveal the positive role of corticosteroids, but most of them used animal models [61] or case series that lacked a control group [22,24]. Therefore, it might be difficult to draw conclusions that corticosteroids have any advantages over nonuse corticosteroids. Besides, although previous studies suggested that the clinical outcome in patients hospitalized with CAP was improved by systemic corticosteroids [13], researchers pointed out that these studies included predominantly CAP cases with bacterial infection and were given appropriate antibiotic therapy; thus, the encouraging results cannot be popularized to all CAP, especially those with viral infection [8].

Nevertheless, our study has some limitations. Firstly, heterogeneity cannot be ignored in our research. Since it is impossible to conduct a clinical trial on critically ill patients, only observational studies were retrieved and enrolled in our meta-analysis. While observational studies are potentially susceptible to bias and induce between-study heterogeneity due to clinical diversity, we cannot draw a robust conclusion. Second, as noticed by clinicians, critical patients were more likely to be given steroids than patients with mild cases [52,62]; the severity of illness should therefore be taken into account. Among the 23 included research papers, 17 studies conducted further multivariable analysis to adjust for potential confounding factors and to determine whether corticosteroid treatment is a predictor of mortality. Among these, 14 studies showed that corticosteroid treatment was not an independent risk factor for mortality, although there was a trend towards greater mortality, while the remaining three studies suggested that corticosteroid treatment could increase the mortality risk independently. Through the reality of the abovementioned factors, we cannot draw a solid conclusion about the effectiveness of corticosteroids in treating severe influenza A (H1N1) cases. Besides, it is difficult to evaluate fairly the effect according to the dose, time given, and baseline of steroid use, because steroid usage was varied by the attending physician and very few studies gave detailed information about it. Hence, further clinical studies – especially those with a comparative and rigorous design regarding the timing, the formulation of corticosteroids, the dosage, the duration, and the length of tapering – as well as randomized studies may help to clarify this issue.

## Conclusions

Our findings suggest that corticosteroids have no beneficial effects in treating patients with influenza A (H1N1). Our results provide evidence regarding the therapeutic strategy for both World Health Organization and USA Centers for Disease Control and Prevention guidelines. A stronger study design and data replication are necessary moving forward.

## Key messages

- Our findings suggest that corticosteroids have no beneficial effects in treating patients with influenza A (H1N1).
- Available evidence did not support the use of corticosteroids as standard care for patients with severe influenza.
- Our paper will be of interest to medical researchers and physicians who fight against influenza A (H1N1) in the first line.

## Additional files

Additional file 1: **Presents the Newcastle-Ottawa Scale for quality assessment.**

Additional file 2: **Presents subgroup analyses for case–control studies.**

Additional file 3: **Presents subgroup analyses for cohort studies.**

Additional file 4: **Presents funnel plots for case–control studies and cohort studies.**

## Abbreviations

CAP: severe community-acquired pneumonia
CI: confidence interval
OR: odds ratio
