# Supplementary material for: Do corticosteroids reduce the mortality of influenza A (H1N1) infection? A meta-analysis
Source: Crit Care. 2015 Dec 1;19:46. doi: 10.1186/s13054-015-0764-5 (PMC4348153; doi:10.1186/s13054-015-0764-5)
Supplement: Supplementary file 1 — Presents the Newcastle-Ottawa Scale for quality assessment. [file 13054_2015_764_MOESM1_ESM.pdf]

# Additional file 1

## Newcastle-Ottawa Quality Assessment Scale: Case-Control Studies

**Selection**

**1 Is the case definition adequate?(*)**

We classified case definition adequately; if the case was confirmed or probable or suspected of Pandemic A(H1N1) 2009 cases and deceased

**2 Representativeness of the cases(*)**

We believe the data is representative if it was surveillance data or multi-center data (multi-center defined as two or more settings). We don't believe the data is representative if it was from one hospital or didn't mention the source of the data.

**3 Selection of controls (*)**

If the controls were from the same population with cases, we gave it one score.

**4 Definition of controls(*)**

We classified control definition adequately, if the control is confirmed or probable or suspected of Pandemic A(H1N1) 2009 cases and patients are alive when the study was conducted.

**Comparability**

**1. Comparability of cases and controls on the basis of the design or analysis(**)**

We believe the two groups were comparable if there were no significant differences between their age(one score) and antiviral use(one score). The scores could not be given if there were significant differences in age or antiviral use, or they were not mentioned.

**Exposure**

**1. Ascertainment of exposure(*)**

We classified exposure certainly if the detail of how corticosteroids use was described in the paper or by the treatment system that was described. We also assessed the source of data. We believe the information was reliable if they were from medical records or structured interviews. People who used steroids for background diseases were excluded from the study.

**2. Same method of ascertainment for cases and controls (*)**

Yes, we gave it one score.

**3. Non-Response Rate (*)**

If the rate was same for both groups, we gave it one score.

We did not score it if non-respondents described or rated it differently with no designation.

<u>**Newcastle-Ottawa Quality Assessment Scale: Cohort Studies**</u>

**Selection**

1. **Representativeness of the exposed cohort(*)**

   We believe the data is representative if 1) all the cases were confirmed or probable or suspected H1N1 cases, and 2) the data were surveillance data or from multi-center data.

2. **Selection of the non-exposed cohort (*)**

   If the non-exposed cohort was drawn from the same community as the exposed cohort, we gave it one score.

3. **Ascertainment of exposure to implants (*)**

   We classified exposure by details of how corticosteroids were administered in the paper and if the information was reported from medical record or structured interview.

4. **Demonstration that outcome of interest was not present at start of study (*)**

**Comparability(**)**

We believe the two groups were comparable if there were no significant differences between their age(one score) and antiviral use(one score). The scores could not be given if there were significant differences in age or antiviral use, or they were not mentioned.

**Outcome**

1. **Assessment of outcome (*)**

   It was assessed if the outcome was from medical record.

2. **Was follow up long enough for outcomes to occur(*)**

   We classified the follow up duration if the data was from retrospective study data or from surveillance data, or if follow up was longer than 16 days.

3. **Adequacy of follow up of cohorts(*)**

   We classified the follow up information if it was complete or subjects that did not follow up introduced further bias(follow up >=80% patients in each group).
