# Supplementary material for: Do corticosteroids reduce the mortality of influenza A (H1N1) infection? A meta-analysis
Source: Crit Care. 2015 Dec 1;19:46. doi: 10.1186/s13054-015-0764-5 (PMC4348153; doi:10.1186/s13054-015-0764-5)
Supplement: Supplementary file 2 — Presents subgroup analyses for case–control studies. [file 13054_2015_764_MOESM2_ESM.pdf]

Additional file 2: Forest plots of subgroup analyses for case control studies

	death		survival			Odds Ratio
Study or Subgroup	Events	Total	Events	Total	Weight	M-H, Random, 95% CI
**2.1.1 sample size(N>=100)**						
Fernando, 2011	38	85	37	93	10.9%	1.22 [0.67, 2.22]
Hong, 2013	62	99	45	146	11.7%	3.76 [2.20, 6.44]
Jose, 2013	29	41	39	88	8.3%	3.04 [1.37, 6.71]
Jung, 2011	54	78	45	141	10.8%	4.80 [2.64, 8.72]
Li, 2012	47	58	436	1071	9.9%	6.22 [3.19, 12.13]
Sertogullarindan, 2011	34	68	12	74	8.5%	5.17 [2.37, 11.27]
Xi, 2010	17	27	35	128	7.5%	4.52 [1.89, 10.81]
Yu, 2011	14	18	40	110	5.0%	6.13 [1.89, 19.88]
Zhang, 2013	236	292	814	1858	15.4%	5.41 [3.98, 7.34]
**Subtotal (95% CI)**		**766**		**3709**	**88.0%**	**4.01 [2.82, 5.71]**
Total events	531		1503			
Heterogeneity: Tau² = 0.17; Chi² = 22.10, df = 8 (P = 0.005); I² = 64%						
Test for overall effect: Z = 7.74 (P < 0.00001)						
**2.1.2 small sample size(N<100)**						
Chawla, 2013	9	10	29	67	1.9%	11.79 [1.41, 98.42]
Kinikar, 2012	9	15	12	77	4.9%	8.13 [2.44, 27.05]
Perez-Padilla, 2009	3	7	2	11	1.9%	3.38 [0.40, 28.74]
Silvio, 2012	4	9	3	11	2.4%	2.13 [0.33, 13.81]
Sun, 2010	7	7	5	11	1.0%	17.73 [0.81, 385.60]
**Subtotal (95% CI)**		**48**		**177**	**12.0%**	**6.21 [2.76, 13.96]**
Total events	32		51			
Heterogeneity: Tau² = 0.00; Chi² = 2.57, df = 4 (P = 0.63); I² = 0%						
Test for overall effect: Z = 4.42 (P < 0.00001)						
**Total (95% CI)**		**814**		**3886**	**100.0%**	**4.22 [3.10, 5.76]**
Total events	563		1554			
Heterogeneity: Tau² = 0.14; Chi² = 25.47, df = 13 (P = 0.02); I² = 49%						
Test for overall effect: Z = 9.09 (P < 0.00001)						
Test for subgroup differences: Chi² = 0.94, df = 1 (P = 0.33), I² = 0%						

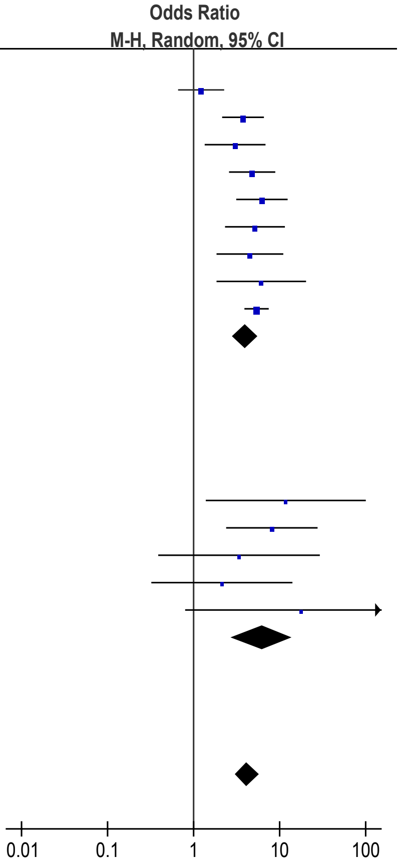

Figure A. Effect of corticosteroid on influenza A (H1N1) cases in different sample size subgroups

Study or Subgroup	death Events	death Total	survival Events	survival Total	Weight	Odds Ratio M-H, Random, 95% CI
**2.2.1 good comparability**						
Chawla, 2013	9	10	29	67	1.9%	11.79 [1.41, 98.42]
Fernando, 2011	38	85	37	93	10.9%	1.22 [0.67, 2.22]
Kinikar, 2012	9	15	12	77	4.9%	8.13 [2.44, 27.05]
Sun, 2010	7	7	5	11	1.0%	17.73 [0.81, 385.60]
Xi, 2010	17	27	35	128	7.5%	4.52 [1.89, 10.81]
Yu, 2011	14	18	40	110	5.0%	6.13 [1.89, 19.88]
Zhang, 2013	236	292	814	1858	15.4%	5.41 [3.98, 7.34]
**Subtotal (95% CI)**		**454**		**2344**	**46.5%**	**4.60 [2.36, 8.98]**
Total events	330		972			
Heterogeneity: Tau² = 0.47; Chi² = 22.38, df = 6 (P = 0.001); I² = 73%						
Test for overall effect: Z = 4.47 (P < 0.00001)						
**2.2.2 poor comparability**						
Hong, 2013	62	99	45	146	11.7%	3.76 [2.20, 6.44]
Jose, 2013	29	41	39	88	8.3%	3.04 [1.37, 6.71]
Jung, 2011	54	78	45	141	10.8%	4.80 [2.64, 8.72]
Li, 2012	47	58	436	1071	9.9%	6.22 [3.19, 12.13]
Perez-Padilla, 2009	3	7	2	11	1.9%	3.38 [0.40, 28.74]
Sertogullarindan, 2011	34	68	12	74	8.5%	5.17 [2.37, 11.27]
Silvio, 2012	4	9	3	11	2.4%	2.13 [0.33, 13.81]
**Subtotal (95% CI)**		**360**		**1542**	**53.5%**	**4.36 [3.28, 5.80]**
Total events	233		582			
Heterogeneity: Tau² = 0.00; Chi² = 3.09, df = 6 (P = 0.80); I² = 0%						
Test for overall effect: Z = 10.10 (P < 0.00001)						
**Total (95% CI)**		**814**		**3886**	**100.0%**	**4.22 [3.10, 5.76]**
Total events	563		1554			
Heterogeneity: Tau² = 0.14; Chi² = 25.47, df = 13 (P = 0.02); I² = 49%						
Test for overall effect: Z = 9.09 (P < 0.00001)						
Test for subgroup differences: Chi² = 0.02, df = 1 (P = 0.89), I² = 0%						

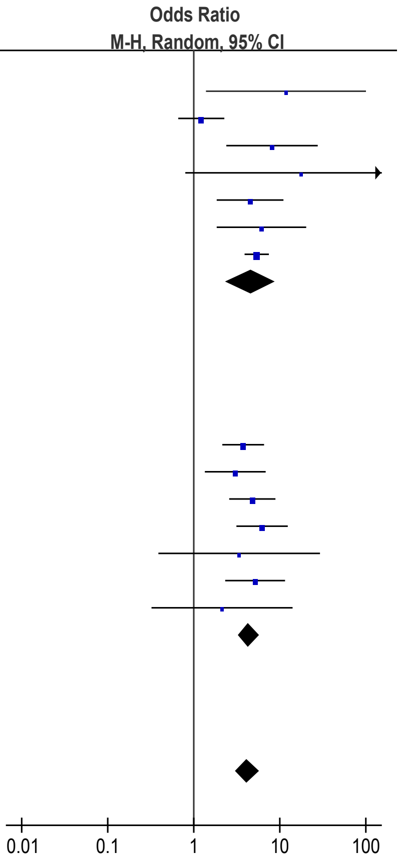

Figure B. Effect of corticosteroid on influenza A (H1N1) cases in subgroups with different comparability between cases and controls

Study or Subgroup	death Events	death Total	survival Events	survival Total	Weight	Odds Ratio M-H, Fixed, 95% CI
**2.3.1 yes**						
Fernando, 2011	38	85	37	93	16.4%	1.22 [0.67, 2.22]
Silvio, 2012	4	9	3	11	1.3%	2.13 [0.33, 13.81]
Sun, 2010	7	7	5	11	0.2%	17.73 [0.81, 385.60]
**Subtotal (95% CI)**		**101**		**115**	**17.9%**	**1.50 [0.87, 2.58]**
Total events	49		45			
Heterogeneity: Chi² = 3.06, df = 2 (P = 0.22); I² = 35%						
Test for overall effect: Z = 1.47 (P = 0.14)						
**2.3.2 no**						
Chawla, 2013	9	10	29	67	0.6%	11.79 [1.41, 98.42]
Hong, 2013	62	99	45	146	11.4%	3.76 [2.20, 6.44]
Jose, 2013	29	41	39	88	6.1%	3.04 [1.37, 6.71]
Jung, 2011	54	78	45	141	8.3%	4.80 [2.64, 8.72]
Kinikar, 2012	9	15	12	77	1.3%	8.13 [2.44, 27.05]
Li, 2012	47	58	436	1071	7.1%	6.22 [3.19, 12.13]
Perez-Padilla, 2009	3	7	2	11	0.7%	3.38 [0.40, 28.74]
Sertogullarindan, 2011	34	68	12	74	4.8%	5.17 [2.37, 11.27]
Xi, 2010	17	27	35	128	3.8%	4.52 [1.89, 10.81]
Yu, 2011	14	18	40	110	2.1%	6.13 [1.89, 19.88]
Zhang, 2013	236	292	814	1858	35.7%	5.41 [3.98, 7.34]
**Subtotal (95% CI)**		**713**		**3771**	**82.1%**	**5.05 [4.14, 6.15]**
Total events	514		1509			
Heterogeneity: Chi² = 4.85, df = 10 (P = 0.90); I² = 0%						
Test for overall effect: Z = 16.09 (P < 0.00001)						
**Total (95% CI)**		**814**		**3886**	**100.0%**	**4.41 [3.67, 5.30]**
Total events	563		1554			
Heterogeneity: Chi² = 25.47, df = 13 (P = 0.02); I² = 49%						
Test for overall effect: Z = 15.86 (P < 0.00001)						
Test for subgroup differences: Chi² = 16.95, df = 1 (P < 0.0001), I² = 94.1%						

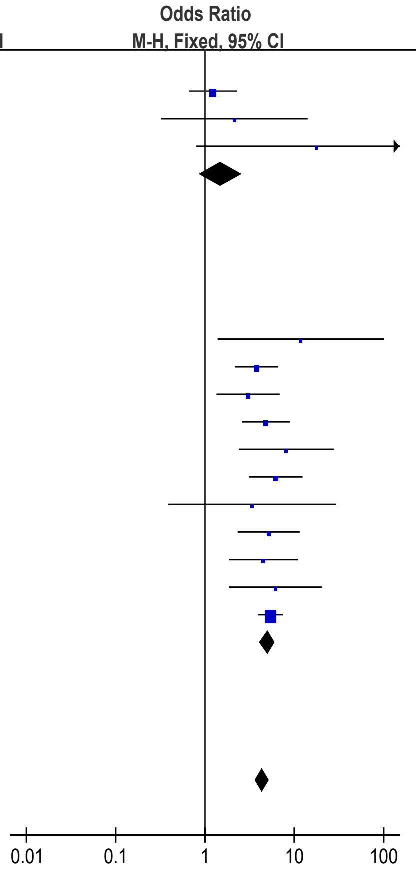

Figure C. Effect of corticosteroid on influenza A (H1N1) cases in subgroups with or without probable and/or suspected Pandemic A(H1N1) cases.
