# Supplementary material for: Do corticosteroids reduce the mortality of influenza A (H1N1) infection? A meta-analysis
Source: Crit Care. 2015 Dec 1;19:46. doi: 10.1186/s13054-015-0764-5 (PMC4348153; doi:10.1186/s13054-015-0764-5)
Supplement: Supplementary file 3 — Presents subgroup analyses for cohort studies. [file 13054_2015_764_MOESM3_ESM.pdf]

Additional file 3: Forest plots of subgroup analyses for cohort studies

Study or Subgroup	steroids Events	steroids Total	non-steroids Events	non-steroids Total	Weight	Risk Ratio M-H, Random, 95% CI
**2.4.1 good quality**						
Diaz, 2012	25	136	41	236	25.3%	1.06 [0.67, 1.66]
Linko,2011	20	43	10	43	14.6%	2.00 [1.06, 3.76]
Christian, 2011	28	83	21	125	22.0%	2.01 [1.23, 3.29]
Viasus,2011	3	37	4	129	3.1%	2.61 [0.61, 11.17]
**Subtotal (95% CI)**		**299**		**533**	**65.0%**	**1.62 [1.09, 2.42]**
Total events	76		76			
Heterogeneity: Tau² = 0.06; Chi² = 4.95, df = 3 (P = 0.18); I² = 39%						
Test for overall effect: Z = 2.39 (P = 0.02)						
**2.4.2 poor quality**						
Zhang,2011	9	109	2	37	3.0%	1.53 [0.35, 6.75]
Ketan, 2013	11	39	3	24	4.7%	2.26 [0.70, 7.28]
I. Martin, 2011	58	126	17	94	23.7%	2.55 [1.59, 4.07]
Chen, 2010	1	6	0	6	0.7%	3.00 [0.15, 61.74]
Mady, 2012	8	72	2	60	2.9%	3.33 [0.74, 15.11]
**Subtotal (95% CI)**		**352**		**221**	**35.0%**	**2.47 [1.66, 3.69]**
Total events	87		24			
Heterogeneity: Tau² = 0.00; Chi² = 0.61, df = 4 (P = 0.96); I² = 0%						
Test for overall effect: Z = 4.44 (P < 0.00001)						
**Total (95% CI)**		**651**		**754**	**100.0%**	**1.85 [1.42, 2.39]**
Total events	163		100			
Heterogeneity: Tau² = 0.02; Chi² = 8.92, df = 8 (P = 0.35); I² = 10%						
Test for overall effect: Z = 4.63 (P < 0.00001)						
Test for subgroup differences: Chi² = 2.12, df = 1 (P = 0.15), I² = 52.9%						

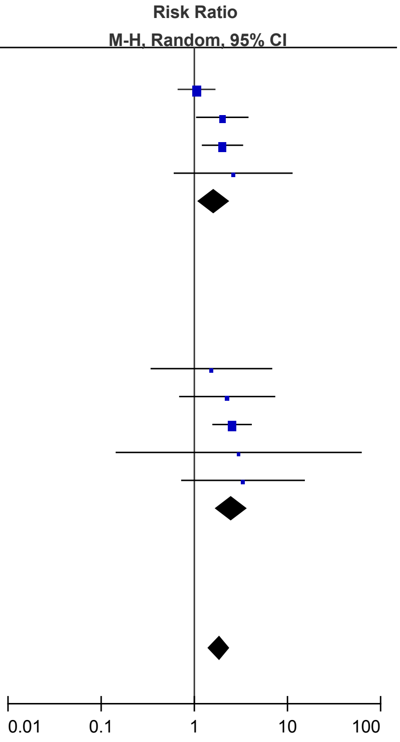

Figure A. Effect of corticosteroid on influenza A (H1N1) cases in subgroups with different study qualities

	steroids		non-steroids			Risk Ratio	Risk Ratio
Study or Subgroup	Events	Total	Events	Total	Weight	M-H, Random, 95% CI	M-H, Random, 95% CI
**2.5.1 good comparability**							
Chen, 2010	1	6	0	6	0.7%	3.00 [0.15, 61.74]	
Christian, 2011	28	83	21	125	22.0%	2.01 [1.23, 3.29]	
Diaz, 2012	25	136	41	236	25.3%	1.06 [0.67, 1.66]	
Linko,2011	20	43	10	43	14.6%	2.00 [1.06, 3.76]	
**Subtotal (95% CI)**		**268**		**410**	**62.7%**	**1.58 [1.06, 2.36]**	
Total events	74		72				
Heterogeneity: Tau² = 0.06; Chi² = 4.63, df = 3 (P = 0.20); I² = 35%							
Test for overall effect: Z = 2.27 (P = 0.02)							
**2.5.2 poor comparability**							
I. Martin, 2011	58	126	17	94	23.7%	2.55 [1.59, 4.07]	
Ketan, 2013	11	39	3	24	4.7%	2.26 [0.70, 7.28]	
Mady, 2012	8	72	2	60	2.9%	3.33 [0.74, 15.11]	
Viasus,2011	3	37	4	129	3.1%	2.61 [0.61, 11.17]	
Zhang,2011	9	109	2	37	3.0%	1.53 [0.35, 6.75]	
**Subtotal (95% CI)**		**383**		**344**	**37.3%**	**2.47 [1.68, 3.65]**	
Total events	89		28				
Heterogeneity: Tau² = 0.00; Chi² = 0.60, df = 4 (P = 0.96); I² = 0%							
Test for overall effect: Z = 4.57 (P < 0.00001)							
**Total (95% CI)**		**651**		**754**	**100.0%**	**1.85 [1.42, 2.39]**	
Total events	163		100				
Heterogeneity: Tau² = 0.02; Chi² = 8.92, df = 8 (P = 0.35); I² = 10%							
Test for overall effect: Z = 4.63 (P < 0.00001)							
Test for subgroup differences: Chi² = 2.47, df = 1 (P = 0.12), I² = 59.5%							

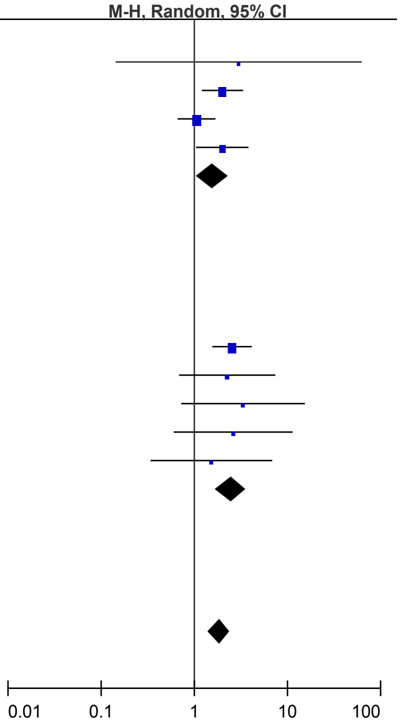

Figure B. Effect of corticosteroid on influenza A (H1N1) cases in subgroup studies with poor and good comparability in terms of age and antiviral treatment

Study or Subgroup	steroids Events	steroids Total	non-steroids Events	non-steroids Total	Weight	Risk Ratio M-H, Fixed, 95% CI
**2.6.1 yes**						
Christian, 2011	28	83	21	125	19.2%	2.01 [1.23, 3.29]
I. Martin, 2011	58	126	17	94	22.3%	2.55 [1.59, 4.07]
**Subtotal (95% CI)**		**209**		**219**	**41.5%**	**2.30 [1.63, 3.23]**
Total events	86		38			
Heterogeneity: Chi² = 0.47, df = 1 (P = 0.49); I² = 0%						
Test for overall effect: Z = 4.78 (P < 0.00001)						
**2.6.2 no**						
Chen, 2010	1	6	0	6	0.6%	3.00 [0.15, 61.74]
Diaz, 2012	25	136	41	236	34.3%	1.06 [0.67, 1.66]
Ketan, 2013	11	39	3	24	4.3%	2.26 [0.70, 7.28]
Linko,2011	20	43	10	43	11.4%	2.00 [1.06, 3.76]
Mady, 2012	8	72	2	60	2.5%	3.33 [0.74, 15.11]
Viasus,2011	3	37	4	129	2.0%	2.61 [0.61, 11.17]
Zhang,2011	9	109	2	37	3.4%	1.53 [0.35, 6.75]
**Subtotal (95% CI)**		**442**		**535**	**58.5%**	**1.53 [1.11, 2.10]**
Total events	77		62			
Heterogeneity: Chi² = 5.42, df = 6 (P = 0.49); I² = 0%						
Test for overall effect: Z = 2.60 (P = 0.009)						
**Total (95% CI)**		**651**		**754**	**100.0%**	**1.85 [1.46, 2.33]**
Total events	163		100			
Heterogeneity: Chi² = 8.92, df = 8 (P = 0.35); I² = 10%						
Test for overall effect: Z = 5.19 (P < 0.00001)						
Test for subgroup differences: Chi² = 2.94, df = 1 (P = 0.09), I² = 66.0%						

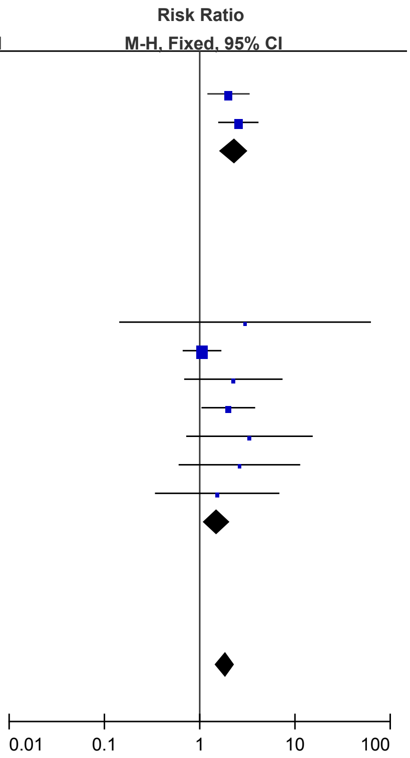

Figure C. Effect of corticosteroid on influenza A (H1N1) cases in subgroups with and without suspected and /or probable cases
