# Supplementary figures and images for: Do corticosteroids reduce the mortality of influenza A (H1N1) infection? A meta-analysis
Source: Crit Care. 2015 Dec 1;19:46. doi: 10.1186/s13054-015-0764-5 (PMC4348153; doi:10.1186/s13054-015-0764-5)

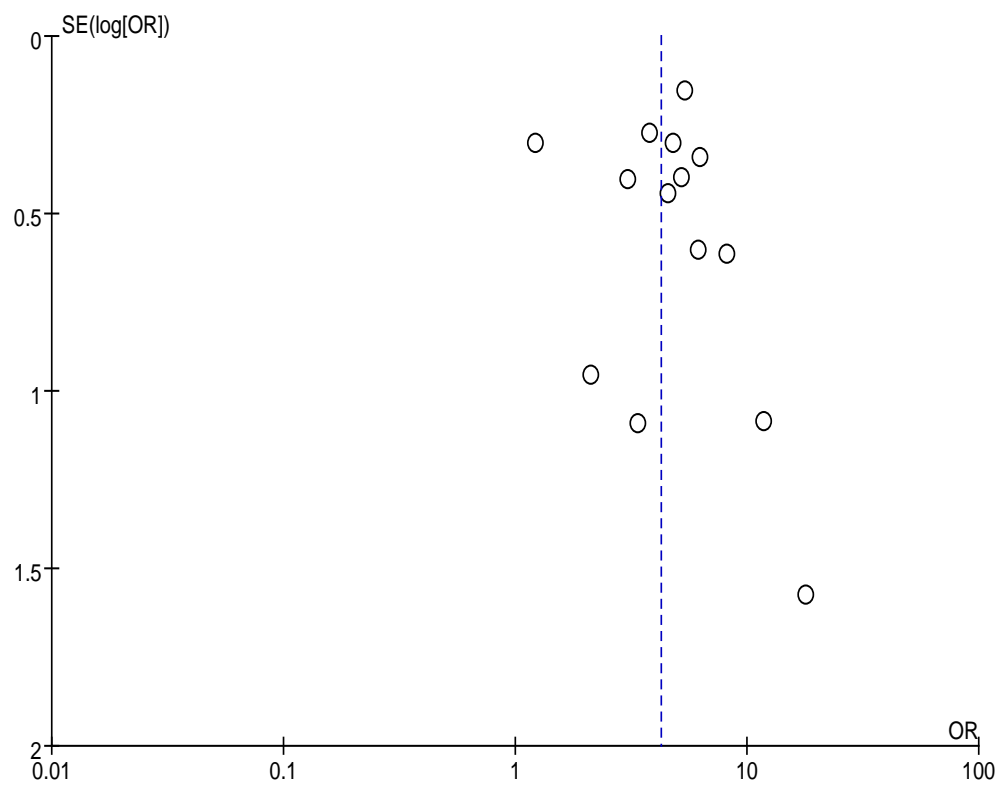

Figure1. Funnel plot for case control studies

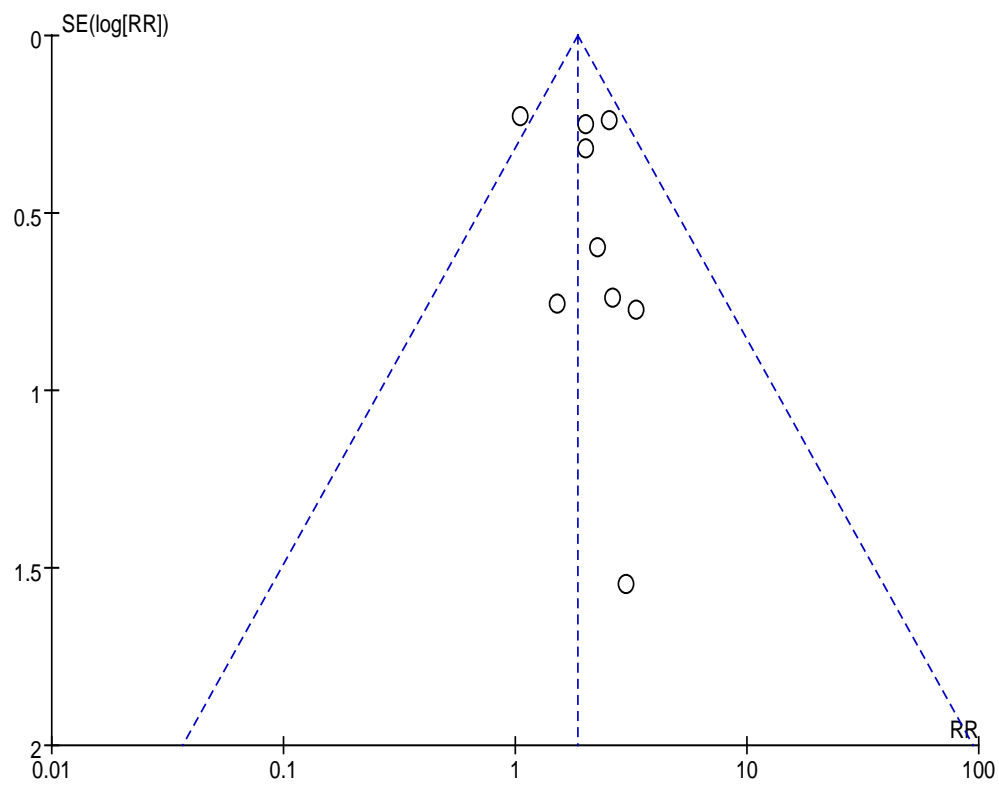

Figure2. Funnel plot for cohort studies

Supplement: Supplementary file 4 — Presents funnel plots for case–control studies and cohort studies. [file 13054_2015_764_MOESM4_ESM.pdf]
